# Prevalence and Antimicrobial Resistance Diversity of *Salmonella* Isolates in Jiaxing City, China

**DOI:** 10.3390/antibiotics13050443

**Published:** 2024-05-14

**Authors:** Ping Li, Li Zhan, Henghui Wang, Yong Yan, Miaomiao Jia, Lei Gao, Yangming Sun, Guoying Zhu, Zhongwen Chen

**Affiliations:** 1 Jiaxing Key Laboratory of Pathogenic Microbiology, Jiaxing Center for Disease Control and Prevention, Jiaxing 314050, China; plijxcdc@163.com (P.L.);; 2 Institute of Microbiology, Zhejiang Provincial Center for Disease Control and Prevention, Hangzhou 310051, China; lzhan@cdc.zj.cn

**Keywords:** prevalence, *Salmonella*, monophonic variant, *S*. Kentucky, clinical diarrhea and food samples, AMR, plasmid replicons, WGS

## Abstract

Nontyphoidal *Salmonella* (NTS) is a cause of foodborne diarrheal diseases worldwide. Important emerging NTS serotypes that have spread as multidrug-resistant high-risk clones include *S.* Typhimurium monophasic variant and *S.* Kentucky. In this study, we isolated *Salmonella* in 5019 stool samples collected from patients with clinical diarrhea and 484 food samples. Antibiotic susceptibility testing and whole-genome sequencing were performed on positive strains. The detection rates of *Salmonella* among patients with diarrhea and food samples were 4.0% (200/5019) and 3.1% (15/484), respectively. These 215 *Salmonella* isolates comprised five main serotypes, namely *S.* Typhimurium monophasic variant, *S.* Typhimurium, *S.* London, *S.* Enteritidis, and *S.* Rissen, and were mainly resistant to ampicillin, tetracycline, chloramphenicol, and trimethoprim/sulfamethoxazole. The MDR rates of five major serotypes were 77.4%, 56.0%, 66.7%, 53.3%, and 80.0%, respectively. The most commonly acquired extended-spectrum β-lactamase-encoding genes were *bla*_TEM−1B_, *bla*_OXA-10_, and *bla*_CTX-M-65_. The *S.* Typhimurium monophasic variant strains from Jiaxing City belonged to a unique clone with broad antibiotic resistance. *S.* Kentucky isolates showed the highest drug resistance, and all were MDR strains. The discovery of high antibiotic resistance rates in this common foodborne pathogen is a growing concern; therefore, ongoing surveillance is crucial to effectively monitor this pathogen.

## 1. Introduction

Nontyphoidal *Salmonella* (NTS), which is one of four causes of foodborne diarrheal diseases worldwide, usually causes self-limiting gastrointestinal infections in humans [1,2]. Outbreaks caused by *Salmonella* infection are a complex public health and economic issue worldwide and necessitate urgent action [3,4]. To date, over 2600 serotypes of *Salmonella* have been identified [5], including more than 2000 NTS serotypes, which predominantly comprise *S*. Enteritidis, *S*. Typhimurium, *S*. Newport, and *S*. Heidelberg [6]. Moreover, invasive NTS (iNTS) causes invasive disease and systemic infections such as bacteremia, meningitis, and other focal infections in children, the elderly, and the immunocompromised [7]. The most predominant iNTS serovars are *S*. Typhimurium, *S*. Choleraesuis, and *S*. Dublin [8,9]. Genetic virulence factors associated with the ability of *S*. Dublin to invade humans’ blood have been well characterized by whole-genome sequencing [10]. A monophasic variant of *S*. Typhimurium (4,[5],12:i:-), which fails to express the second-phase flagellar antigen FljB, has emerged as a major global cause of NTS disease in animals and humans [11,12,13].

Antimicrobial resistance (AMR) is a global public health concern. *Salmonella* is one microorganism in which resistant serotypes have emerged because of the widespread use of antibiotics in the production of food animals as well as the indiscriminate use of antibiotics in clinics [14]. Commonly isolated serotypes, including *S*. Enteritidis, *S*. Typhimurium, *S*. Newport, and *S*. Typhimurium monophasic variant, have shown higher rates of AMR compared with minor serotypes of *Salmonella* infections from many countries [15,16]. CTX-M and TEM have been reported as the predominant types of extended-spectrum beta-lactamase (ESBL) enzymes in *Salmonella* [17]. ESBL-producing *Salmonella* isolates confer resistance to some of the antibiotics commonly used in humans, including third-generation cephalosporins [18]. Phage therapy was considered as an alternative to antibiotics for the treatment of antibiotic-resistant bacterial infections. Genomic analysis of Anderson typing phages of *S.* Typhimrium was used to understand the complex dynamics of bacteria–phage interaction through characterizing the genetic determinants that are responsible for their differing host ranges [19].

The *S*. Typhimurium monophasic variant (4,[5],12:i:-) serotype emerged from *S*. Typhimurium (4,[5],12:i:2) and is characterized by its lack of expression of the second-phase flagellar antigen FljB [20]. Since its identification in poultry in the late 1980s [21], isolates of 4,[5],12:i:- have spread rapidly and have been reported in various countries at different times [22,23]. Studies of *S*. Typhimurium monophasic variant strains have found that most are multidrug-resistant [11,24,25]. Tn6029 transposon encodes resistance to ampicillin (*bla*_TEM-1_), sulfonamides (*sul*2), and streptomycin (*aph(3”)-Ib* and *aph(6)-Id*) together with the *tet*(B) gene carried on Tn*10*, which contributes to the ampicillin, streptomycin, sulfonamide, and tetracycline (ASSuT) resistance profile of *Salmonella* 4,[5],12:i:-. Moreover, the *tet*(B) as well as remaining ASSuT genes could be integrated into the chromosome of *S*. Typhimurium monophasic variant strains. In contrast, the ASSuT profile was plasmid-mediated for *tet*(A)-carrying monophasic *S*. Typhimurium isolates [26]. Furthermore, chromosome- and plasmid-mediated colistin resistance have also been reported in *Salmonella* 4,[5],12:i:- isolates [27]. 

The high rates of prevalence and AMR have impacted public health. *S*. Kentucky sequence type (ST)198 is mostly isolated from chickens and humans [28,29]. *bla*_CTX-M-55_, *rmtB*, *tet*(A), *floR*, and *fosA*3, together with amino acid substitution in *gyrA* (S83F and D83N) and *parC* (S80I), have contributed to the high resistance to ciprofloxacin, cephalosporin, and fluoroquinolones in *S*. Kentucky isolates [30,31,32]. The IncHI2 plasmid carries numerous resistance genes including *bla*_CTX-M_, *aadA7*, *lnu*(F), *bla*_TEM-1b_, *rmtB*, and *mph*(A), except for *bla*_CTX-M-14b_, which is inserted into the chromosomes of *S*. Kentucky isolates, enabling them to transfer vertically as intrinsic chromosomal genes within this lineage. *bla*_CTX-M-14b_ in *S*. Kentucky ST198 also has been reported as chromosomally located [33]. The *S*. Kentucky ST198 could be genetically divided into two clades, namely ST198.1 and ST198.2. Moreover, the clade ST198.2 contains two subclades, namely ST198.2-1 and ST198.2-2, in China. Co-existence of the ESBL-encoding genes *bla*_CTX-M-55_ and *bla*_TEM-1b_ in ST198.2-2 is one of the characteristics of resistance genes different from the *bla*_CTX-M-14b_ in ST198.2-1 [17].

This study aimed to analyze the prevalence, AMR, and genetic characteristics of 215 clinical and foodborne *Salmonella* isolates collected from Jiaxing City in 2023. Our findings provide a basis for the prevention and control of AMR in *Salmonella*.

## 2. Results

### 2.1. Salmonella Prevalence and Serotypes

A total of 215 *Salmonella* isolates were obtained in 2023 including 200 from clinical samples of patients with diarrhea and 15 from food samples. Of the 35 serotypes identified, the top five were *S*. Typhimurium monophasic variant, *S*. Typhimurium, *S*. London, *S*. Enteritidis, and *S*. Rissen, accounting for 39.1%, 11.6%, 7.0%, 7.0%, and 4.7%, respectively.

**Figure 1:**
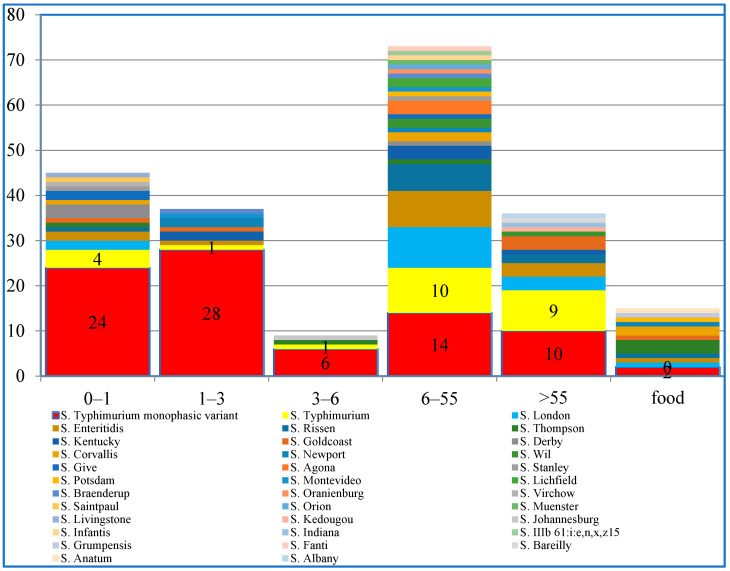
Serotype distribution of *Salmonella* isolates in different groups.

The detection rate of *Salmonella* among patients with diarrhea was 4.0% (200/5019). Among the 5019 cases of diarrhea reported, the ratio of males to females was 1.09:1, and the 21- to 30-year-old age group was most common. Children under the age of 10 accounted for 9.1%. Of the 200 confirmed cases of *Salmonella* infection, 122 were male and 78 were female. The median age of infected patients was 26 years (range: 1 month to 81 years). Predominant symptoms included diarrhea (98.2%), abdominal pain (36.8%), and vomiting (26.5%). Few (9.4%) had fever. *S*. Typhimurium monophasic variant was the predominant serotype in all age groups, mainly in children < 6years. Serotype diversity was highest in cases aged 6–55 years. A total of 24 serotypes were characterized in this group (Table S1 and Figure 1).

Fifteen *Salmonella* isolates were collected from food samples. The overall percentage of food-positive samples was 3.1% (15/484). Six (6/15) were isolated from raw animal meat, including fresh pork and frozen mutton. Five (5/15) were from freshwater animal products, namely fresh carp, live grass carp, live bass, and Bellamya quadrata. Two isolates each were from seasoned raw meat (2/15) and Chinese salad (2/15). The top three serotypes of the foodborne isolates were *S*. Thompson, *S*. Corvallis, and *S*. Typhimurium monophasic variant (Table S1). 

### 2.2. Phenotypic AMR Patterns

Using antibiotic susceptibility tests, we determined that more than half of the isolates were resistant to ampicillin (158/215, 73.5%), tetracycline (149/215, 69.3%), chloramphenicol (124/215, 57.7%), and trimethoprim/sulfamethoxazole (110/215, 51.2%). Only 29 isolates (22.9%) demonstrated susceptibility to all 22 antibiotics tested (Table 1). Over half of the isolates (134/215, 62.3%) were multidrug resistance (MDR). All isolates of *S*. Kentucky and nine other rare serotypes had MDR. All isolates belonged to nine rare serotypes that exhibited MDR, probably because of the small number of them. The percentage of MDR in *S*. Rissen, *S*. Typhimurium monophasic variant, *S*. Derby, *S*. London, *S*. Goldcoast, *S*. Agona, *S*. Typhimurium, *S*. Enteritidis, and *S*. Thompson were 80%, 77.4%, 75.0%, 66.7%, 66.7%, 66.7%, 56.0%, 53.3%, and 50%, respectively (Table 2). *S*. Typhimurium monophasic variant isolates, the core serotype among patients, exhibited high rates of resistance to tetracycline, ampicillin, chloramphenicol, trimethoprim/sulfamethoxazole, and ampicillin/sulbactam (96.4%, 94.1%, 73.8%, 64.3%, and 56.0%, respectively). Over half of *S*. Typhimurium isolates showed resistance to ampicillin, tetracycline, chloramphenicol, and trimethoprim/sulfamethoxazole. The highest antibiotic resistance rates were observed in the six *S*. Kentucky isolates, all of which were resistant to tetracycline, ampicillin, cefazolin, cefotaxime, ciprofloxacin, and nalidixic acid. Most (5/6) of the *S*. Kentucky isolates were also resistant to chloramphenicol and trimethoprim/sulfamethoxazole (Table 1). The two *S*. Kentucky isolates belonging to subclade ST198.2-2 exhibited additional resistance to azithromycin, gentamicin, and ceftazidime, in contrast to the four subclade ST198.2-1 isolates (Table S2). Rates of resistance to chloramphenicol, tetracycline, ampicillin, and trimethoprim/sulfamethoxazole among the 15 foodborne isolates were 60.0%, 53.3%, 40.0%, and 40.0%, respectively. 

### 2.3. AMR Genes and Plasmid Replicons

Next, AMR genotyping was performed using ResFinder 4.1. As shown in Tables S1 and S3, we identified 71 different AMR genes in the 215 *Salmonella* strains. Apart from ESBL-encoding genes and AmpC β-lactamases, the major forms of AMR were the chloramphenicol resistance gene *floR* in 92 isolates (92/215), the sulfonamide resistance gene *sul2* (91/215), and the quinolone resistance gene *qnrS1* (81/215). The fosfomycin resistance gene *fosA7* was exclusively found in the chromosomes of each isolate of *S*. Agona (3/3), *S*. Derby (4/4), and *S*. Grumpensis (1/1). There were 48 and 29 distinct resistant genes detected in *S*. Typhimurium monophasic variant and *S*. Typhimurium isolates, respectively (Table S3). The most prevalent AMRs in monophasic *S*. Typhimurium were *bla*_TEM-1B_ followed by *tet*(B), *sul2*, and *qnrS1*. The top three AMRs carried by *S*. Typhimurium were *bla*_TEM-1B_, *floR*, and *sul2*. However, the proportion of *aph(6)-Id*, *aph(3″)-Ib*, and *tet*(B) in *S*. Typhimurium monophasic variant is much higher than that in *S*. Typhimurium. The tetracycline efflux pump gene *tet*(B) was only found in monophasic *S*. Typhimurium strains. The prevalences of lincosamide resistance gene *lnu*(F), trimethoprim resistance gene *dfrA14*, ESBL-encoding genes such as *bla*_OXA-10_ and *bla*_CTX-M-65_, and rifampin resistance gene *arr-2* were much higher in *S*. Typhimurium monophasic variant isolates than in *S*. Typhimurium and other serotype isolates. In contrast, *tet*(A) and *tet*(M) were more prevalent in *S*. Typhimurium compared to its monophasic variant (Tables S1 and S3).

**Figure 2:**
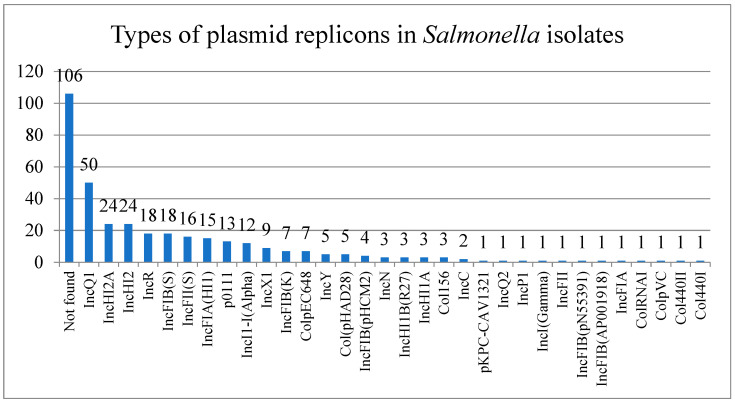
Types of plasmid replicons in *Salmonella* isolates.

Almost half of (109/215) the *Salmonella* isolates were found to be positive for plasmid replicons’ sequence. A total of 32 types of plasmid replicons were detected, with IncQ1 being the most abundant (*n* = 50), followed by IncHI2/IncHI2A (*n* = 24) and IncR (*n* = 18) (Figure 2). Also, some types of plasmids exhibited perfect correspondence to the serotypes; for example, IncQ1 was only detected in *S*. Typhimurium monophasic variant isolates, and IncFIB(S)/IncFII(S)/IncX1 was mainly detected in *S*. Enteritidis. IncHI1A/IncHI1B was only found in *S*. Goldcoast isolates. The most commonly acquired ESBL-encoding genes detected among these strains were *bla*_TEM−1B_ (120/215), followed by *bla*_OXA-10_ (23/215) and *bla*_CTX-M-65_ (21/215). Four *bla*_CTX-M_ subtypes (*bla*_CTX-M-55_, *bla*_CTX-M-14_, *bla*_CTX-M-65_, and *bla*_CTX-M-24_), three *bla*_OXA_ subtypes (*bla*_OXA-1_, *bla*_OXA-10_, and *bla*_OXA-16_), and two *bla*_TEM_ subtypes (*bla*_TEM-1B_ and *bla*_TEM-1A_) were identified among these strains. One *S*. Kedougou isolate and one *S*. Typhimurium monophasic variant were found to be positive for *bla*_LAP-2_. The *bla*_DHA-1_ and *bla*_CARB-2_ were carried by one *S*. Stanley isolate and one *S*. Albany isolate, respectively. In addition, mobile colistin resistance gene *mcr-1.1* was detected along with *bla*_TEM−1B_ and *bla*_CTX-M-14_ in one *S*. Typhimurium monophasic variant strain (Table S1).

### 2.4. Chromosomal Point Mutation in the Quinolone Resistance-Determining Regions

In total, 106 isolates exhibited corresponding mutations in the *parC* gene and/or *gyrA* gene. Two-point mutations occurred within both the *parC* gene and *gyrA* gene for all *S*. Kentucky isolates, namely Ser (83) to Phe and Ser (83) to Tyr within the *gyrA* gene together with Ser (80) to Ile and Thr (57) to Ser for the *parC* gene. Apart from *S*. Kentucky isolates, there were 76 isolates that exhibited a mutation from Thr (57) to Ser in the *parC* gene. Within *gyrA*, 14 isolates presented one replacement in amino acid 83, with three having a change from Ser (83) to Phe, and the other 11 were from Ser (83) to Tyr. Also, there were 17 isolates that presented one replacement in *gyrA* in amino acid 87 with only one isolate having a change from Asp (87) to Gly and the other 16 being from Asp (87) to Tyr. Mutations occurred only within *gyrA* for all *S*. Enteritidis isolates. Most of them had a p.D87Y mutation, while only two isolates exhibited p.S83Y mutation. Most *S*. Typhimurium monophasic variant isolates had mutations neither in *gyrA* nor in *parC* (Table S1).

### 2.5. Phylogenetic Analysis

Using whole-genome sequencing (WGS) analysis, 39 distinct STs were identified among the 215 *Salmonella* isolates, with the predominant ST34 accounting for 39.1% (84/215), followed by ST19 at 11.6% (25/215), ST11 at 7.0% (15/215), ST155 at 7.0% (15/215), and ST469 at 4.7% (10/215). Remarkably, some of the STs exhibited perfect correspondence to the serotypes, including ST34 for *S*. Typhimurium monophasic variant and ST155 for *S*. London. There were also pairs of STs that corresponded to the same serotype, including ST654 and ST516 for *S*. Give, ST2529 and ST358 for *S*. Goldcoast, ST2039 and ST408 for *S*. Potsdam, and ST166 and ST46 for *S*. Newport.

**Figure 3:**
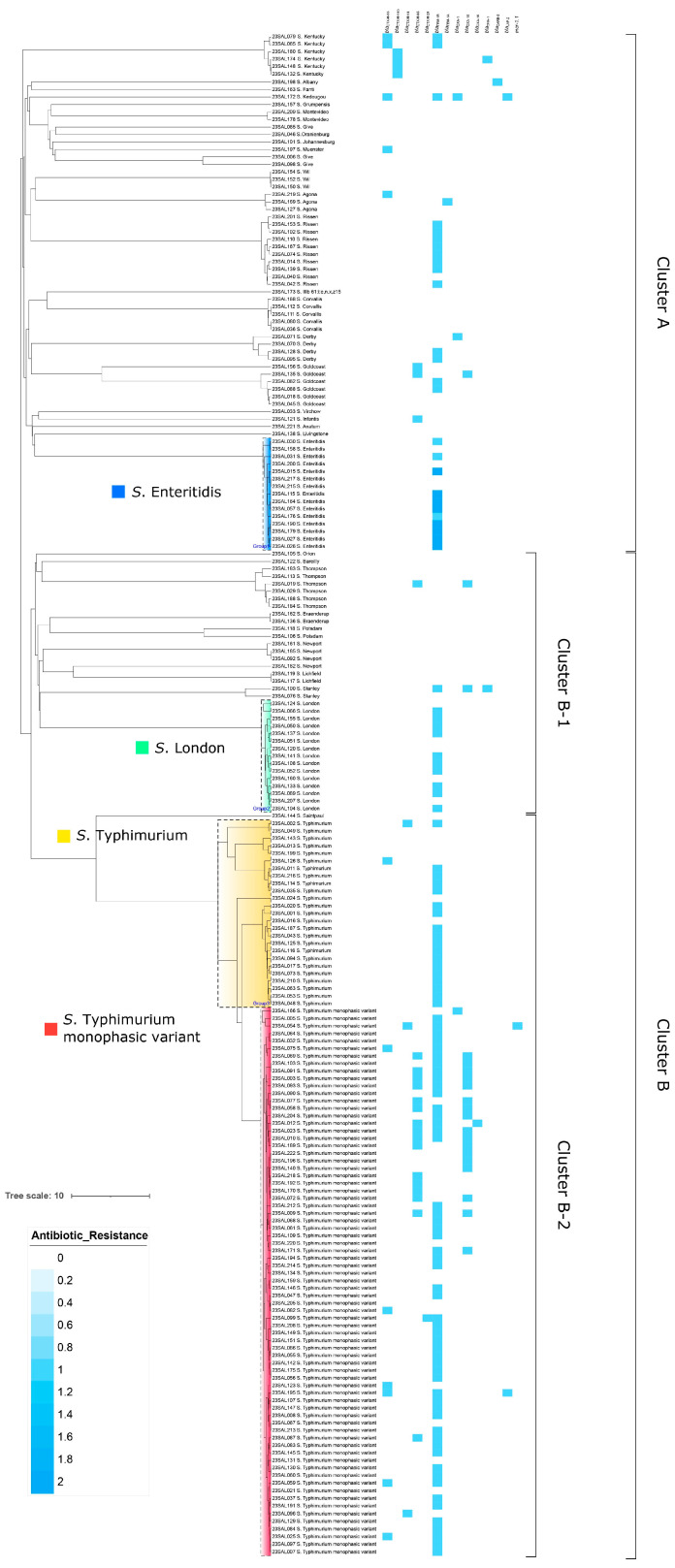
Maximum likelihood tree of 203 *Salmonella* isolates in this study. Blue boxes indicate the presence of ESBLs and colistin resistance genes (*bla*_CTX-M-55_, *bla*_CTX-M-14b_, *bla*_CTX-M-14_, *bla*_CTX-M-65_, *bla*_CTX-M-24_, *bla*_TEM-1B_, *bla*_TEM-1A_, *bla*_OXA-1_, *bla*_OXA-10_, *bla*_OXA-16_, *bla*_DHA-1_, *bla*_CARB-2_, *bla*_LAP-2_, *mcr-1.1*).

To assess genetic variation among these isolates, we conducted phylogenetic analysis of the 203 isolates. As shown in Figure 3, all isolates were classified into two distinct clusters. Cluster A comprised 69 isolates belonging to 24 different STs: ST198, ST292, ST10035, ST1543, ST2060, ST4, ST654, ST23, ST515, ST321, ST516, ST8334, ST13, ST469, ST10422, ST1541, ST40, ST2529, ST358, ST16, ST32, ST64, ST543, and ST11. Cluster B included 134 isolates, representing 14 distinguishable STs: ST684, ST203, ST26, ST22, ST2039, ST408, ST166, ST46, ST214, ST29, ST155, ST49, ST19, and ST34. Cluster B was further subdivided into B-1 and B-2. Cluster B-2 contained all *S*. Typhimurium monophasic variant and *S*. Typhimurium isolates, as well as one *S*. Saintpaul isolate. Interestingly, 15 of the *S*. Typhimurium isolates shared more similarities with five *S*. Typhimurium monophasic variant isolates than with 10 of the other *S*. Typhimurium strains, suggesting a potential genetic relatedness between these isolates.

## 3. Discussion

In this study, we determined the prevalence and genetic diversity of *Salmonella* strains isolated from clinical and food samples collected in Jiaxing City, China. The detection rate of *Salmonella* among individuals with diarrhea was 3.98% (200/5019), which was consistent with previous findings obtained in Ethiopia and lower than that in Shanghai [34,35]. Several studies have demonstrated the dominant prevalence of monophasic *S*. Typhimurium in human, food, and environmental samples [36,37]. Similar to previous studies, *S*. Typhimurium monophasic variant 4,[5],12:i:- was the top serotype among our patients with diarrhea, especially among the younger age groups [38]. Among the foodborne isolates, we identified *S*. Thompson, *S*. Corvallis, and *S*. Typhimurium monophasic variant as the three major serotypes, in contrast to *S*. Typhimurium conducted in Huzhou district, Zhejiang province [39].

*Salmonellosis* outbreaks are linked to the consumption of contaminated food products, including broiler meat, pork, egg, chocolate, and chocolate products [40,41]. The percentage of *Salmonella*-positive samples (10.4%) in freshwater animal products was higher in the present study than in other studies [42]. Marine products including fresh carp, live grass carp, live bass, and Bellamya quadrata have been shown to be contaminated by *Salmonella*, indicating that aquatic products remain an important reservoir of *Salmonella* contamination and possible fecal contamination [43]. NTS outbreaks associated with chocolate consumption have occurred in multiple countries [44,45]. However, this type of food was not positive for NTS in our study. 

A variety of ESBL genes, including *bla*_TEM_, *bla*_CTX-M_, *bla*_SHV_, *bla*_ACC_, and *bla*_CMY_, could be carried by *Salmonella* isolates [46]. In this study, we had found a wide distribution of the ESBL-encoding gene *bla*_TEM-1B_ in different serotypes of *Salmonella* isolates. The *bla*_CTX-M-55_ type is mediated by MDR IncA/C2 and IncHI2 plasmids in *S. enterica* from pork and fish samples [47]. A recent study also demonstrated the cross-species dissemination of the *bla*_CTX-M-55_-positive IncHI2 plasmid by chromosome–plasmid conjugation [48]. In this study, *bla*_CTX-M-55_ was found in *S*. Agona, *S*. Kentucky, *S*. Typhimurium, and *S*. Typhimurium monophasic variant, as well as minor serotypes such as *S*. Kedougou and *S*. Muenster, whilst some of them were positive for plasmid replicon sequences. Other β-lactamase such as *bla*_CARB-2_, *bla*_LAP-2_, and *bla*_DHA-1_ were also identified in *Salmonella* isolates. *bla*_LAP-2_ was flanked by mobile elements (*IS*26, *IS*As17, and *IS*Kpn19) which could cluster and be combined with resistance genes of plasmids [49]. One *S*. Albany isolate was positive for *bla*_CARB-2_ as well as *tet*(G) in this study. This feature seems common in *S*. Typhimurium strains in Mexico, since genomic island 1 harbors a class-1 integron containing multiple gene cassettes (i.e., *aadA2*, *bla_CARB-2_*, *floR*, *sul1*, *tet(G)*) [50]. DHA-1, belonging to class C β-lactamases, was identified in *S*. Montevideo and *S*. Indiana isolates of clinical and animal origins [51,52]. We found one *S*. Stanley isolate that carried *bla*_DHA-1_ together with other resistance genes including *bla*_TEM-1B_, *bla*_OXA-10_, *msr*(E), and *floR*. Plasmid replicons like IncHI2/IncHI2A, IncN, and IncR were identified in this isolate.

Previous studies reported that resistance genes are consistent with the antibiotic resistance phenotypes of the S. 1,4,[5],12:i:- in *aac(3)-IV*, *aac(6′)-Iaa*, *bla*_TEM_, *bla*_CTX-M_, *sul*, *tet*, and *mcr*, as well as plasmid-mediated quinolone resistance genes like *oqxAB*, *qnrS*, and *qnrB* [53]. In this study, various horizontally acquired quinolone-resistant genes (*qnrS1*, *qnrB6*, *qnrS2*, *qnrB4*, *qnrB19*, *aac(6′)-Ib-cr*, and *oqxAB*) were identified in 106 isolates. Over half of them (64/106) conferred resistance to nalidixic acid and/or ciprofloxacin. A total of 19 isolates were resistant to azithromycin. Four azithromycin resistance determinant profiles were identified in 10 isolates, namely *mph*(A) alone (*n* = 7), *mph*(A)–*mph*(E)–*msr*(E)–*erm*(B) (*n* = 1), *mph*(A)–*mph*(E)–*msr*(E) (*n* = 1), and *mph*(A)–*erm*(B) (*n* = 1). Only one *S*. Typhimurium monophasic variant isolate was found to be positive for a horizontally acquired colistin-resistant gene (*mcr*), but three *S*. Enteritidis isolates and one *S*. Typhimurium monophasic variant isolate showed resistance to colistin and polymyxin. Chromosomally encoded polymyxin-resistant traits might account for its polymyxin resistance phenotype [54]. The inconsistencies between resistance phenotypes and resistance genes require further comprehensive elucidation at the genetic level.

Isolates of *S*. Typhimurium monophasic variant, the most prevalent serotype in this study, tend to develop resistance against commonly prescribed antibiotics more frequently than those of *S*. Typhimurium and other minor serotypes [55]. The prevalence of *tet*(A) and *tet*(B) in ST34 *Salmonella* 4,[5],12:i:- isolates indicates that there were at least two lineages of 4,[5],12:i:- circulating in Jiaxing City. Reports of resistance to third-generation cephalosporins and colistin in *Salmonella* 4,[5],12:i:- worldwide have become a cause for serious concern in human medicine [27,36,56]. Sequence types of *S*. Typhimurium lineages including ST19, ST34, ST36, and ST313 have been characterized. Among them, monophasic variants of *S*. Typhimurium have been detected in ST34 and ST313 [57]. In our study, all strains of *S*. Typhimurium belonged to ST19, in contrast to ST34 for monophasic *S*. Typhimurium isolates. New strains of monophasic *S*. Typhimurium appear to be continuously emerging from different *S*. Typhimurium strains via different genetic events [26]. The phylogeny analysis in this study revealed that 84 *S*. Typhimurium monophasic variant stains, together with 10 *S*. Typhimurium strains, comprised a unique clone with broad antibiotic resistance. 

*S*. Kentucky is the most prevalent *Salmonella* serovar in chicken and associated samples [17]. Notably, the prevalence of *S.* Kentucky ST198 (6/200 human isolates, 3.0%) in this study was much higher than that among *Salmonella* strains isolated from patients, poultry, and meat products in another study conducted in China from 2013 to 2017 (0.18%) [29]. Ciprofloxacin is a third-generation quinolone used to treat *Salmonella* infection in immunocompromised patients [58]. It is worth mentioning that all *S*. Kentucky isolates in this study were resistant to ciprofloxacin. Of note, the plasmid-mediated *qnrS1* gene, which confers reduced susceptibility to ciprofloxacin, was carried by two ST198.2-2 *S*. Kentucky isolates. Point mutations in quinolone-resistance-determining regions (QRDRs) in *gyrA* (p.S83F and p.D87G) and *parC* (p.T57S and p.S80I) resulted in resistance to nalidixic acid and ciprofloxacin for all *S*. Kentucky isolates. 

## 4. Materials and Methods

### 4.1. Sample Collection

A total of 5019 stool samples from patients with clinical diarrhea (three or more episodes of diarrhea within 24 h, watery or sticky stools, mucus or pus-bloody stools) were collected to isolate *Salmonella* spp. from 1 January 2023, to 31 December 2023. All fecal samples were cultured overnight at 37 °C in local hospitals using Columbia Blood Agar Plates (Chromagar, Paris, France). Suspected colonies were further confirmed by Matrix-assisted Laser Desorption/Ionization Time-of-Flight Mass Spectrometry (MALDI-TOF MS). All *Salmonella* isolates were submitted to the laboratory of Jiaxing Center for Disease Control and Prevention for further validation and serotyping.

Food samples of raw animal meat (48/484), seasoned raw meat (48/484), prepackaged refrigerated cooked meat products (48/484), freshwater products of animals (48/484), chocolate and chocolate products (48/484), cold-prepared ready-to-eat food (192/484), bean products (20/484), and edible fungus (32/484) were collected and forwarded for *Salmonella* spp. testing according to the GB 4789.4-2016 “National Food Safety Standard for Microbiological Examination of Food–Salmonella Examination” [59]. Briefly, 25 g food samples were placed in 225 mL of buffered peptone water (BPW) (Hopebio, Qingdao, China) enrichment solution and cultured at 36 °C for 18 h. Then, 1 mL of BPW enrichment solution was pipetted into 10 mL of tetrathionate broth (TTB) (Hopebio, Qingdao, China), and 10 mL of selenite cultures was streaked onto xylose lysine deoxycholate (XLD) agar (Hopebio, Qingdao, China) and chromogenic Salmonella agar (Chromogar, Paris, France) and incubated at 36 °C for 24 to 48 h. Suspicious colonies were picked and subjected to biochemical identification using Vitek2 Compact (Biomerieux, Craponne, France). 

### 4.2. Serotyping

Serotyping for the identification of somatic antigen O and flagellar antigens H (phase 1 and 2) was performed by the slide agglutination method according to the White–Kaufmann–Le Minor Scheme. Monophasic *S*. Typhimurium (4,[5]:i:-) was confirmed by PCR assay according to the characterization of the *fliB*–*fliA* intergenic region and phase 2 (*fliB*) flagellar gene.

### 4.3. Antimicrobial Susceptibility Testing

Antimicrobial susceptibility testing was performed using the microdilution method as recommended by the Clinical and Laboratory Standards Institute guidelines (CLSI, 2023). The following antimicrobial agents were tested: nalidixic acid (NAL, 2–64 μg/mL), ciprofloxacin (CIP, 0.015–32 μg/mL), ampicillin (AMP, 1–64 μg/mL), ampicillin/sulbactam (AMS, 1/0.5–64/32 μg/mL), cefotaxime (CTX, 0.25–16 μg/mL), ceftazidime (CAZ, 0.5–32 μg/mL), cefoxitin (CFX, 0.5–32 μg/mL), cefazolin (CFZ, 0.25–32 μg/mL), azithromycin (AZM, 2–64 μg/mL), gentamicin (GEN, 1–32 μg/mL), chloramphenicol (CHL, 2–64 μg/mL), imipenem (IMP, 0.25–8 μg/mL), tetracycline (TET, 1–32 μg/mL), colistin (CT, 0.12–8 μg/mL), trimethoprim/sulfamethoxazole (SXT, 0.5/9.5–8/152 μg/mL), polymyxin (POL, 0.12–4 μg/mL), amikacin (AMI, 4–64 μg/mL), meropenem (MEM, 0.12–4 μg/mL), ertapenem (ETP, 0.25–8 μg/mL), amoxicillin/clavulanic acid (AMC, 2/1–64/32 μg/mL), ceftazidime (CZA, 0.25/4–8/4 μg/mL), cefepime (CPM, 1–32 μg/mL), streptomycin (STR, 4–32 μg/mL), and amoxicillin/clavulanic acid (AMC, 2/1–64/32 μg/mL). Minimum inhibitory concentration (MIC) was interpreted by CLSI breakpoints (CLSI M100, 2023) [60]. ATCC 25922 was used as quality control strain for susceptibility testing.

### 4.4. Whole-Genome Sequencing and Bioinformatics Analyses

Genomic DNA was extracted using the QIAamp DNA mini kit (Qiagen, Hilden, Germany) following the manufacturer’s protocol. Libraries were prepared for Illumina pair-end sequencing using the NEB Next Ultra DNA Library Prep Kit for Illumina (New England Biolabs, Ipswich, MA, USA) and sequenced in a NextSeq 550 sequencer (Illumina platform) (Illumina, San Diego, CA, USA) (150-bp paired-end reads with about >100-fold average coverage). Reads were assembled using SPAdes 3.6. 

### 4.5. Genomic Analysis

Serovars assigned from Kauffmann–White scheme were confirmed by SeqSero2 v1.1.1 [61]. Annotation of mobile elements was carried out using online databases such as ISfinder [62]. Plasmid replicons and antibiotic resistance genes were detected by uploading the assembled data to online databases found in Center for Genomic Epidemiology (https://www.genomicepidemiology.org/, accessed on 30 March 2024.) (PlasmidFinder 2.1 and ResFinder 4.1, respectively). Chromosomal mutations mediating AMR in *acrB*, *pmrA*, *pmrB*, *gyrA*, *gyrB*, *parC*, *parE*, and 16S-*rrsD* were detected by BLAST. In silico 7-gene MLST was performed using the Sequence query tool implemented in the PubMLST Salmonella database. Full genomes of 215 isolates were compared based on their nucleotides composition and sequences by using the BioNumerics software version 7.6 (Applied Maths, Sint-Martens-Latem, Belgium). Forward and reverse fastq files were imported, and the quality of the individual reads was analyzed. A total of 203 sequences were subjected to phylogenetic analysis. A similarity matrix was calculated and used to construct a dendrogram based on the unweighted pair group method (UPGM). The visualization and annotation of the phylogenetic tree were carried out using Itol [63].

### 4.6. Nucleotide Sequence Accession Numbers

The genomes of the 203 *Salmonella* isolates reported in this study have been deposited in the National Center for Biotechnology Information and registered as BioProject numbers PRJNA1109708, PRJNA1109717, PRJNA1109739, PRJNA1109748, PRJNA1109751, PRJNA1109762, PRJNA1109925, PRJNA1109927, PRJNA1109930, PRJNA1109934, PRJNA1109983, PRJNA1109997, PRJNA1110000, PRJNA1110008, and PRJNA1110012.

## 5. Conclusions

In conclusion, we identified 215 *Salmonella* isolates from clinical diarrhea and food samples collected from Jiaxing City, China. *S.* Typhimurium monophasic variant, *S.* Typhimurium, *S.* London, *S.* Enteritidis, and *S.* Rissen were the most prevalent serotypes among these positive isolates. The majority of *Salmonella* isolates were mainly resistant to ampicillin, tetracycline, chloramphenicol, and trimethoprim/sulfamethoxazole. *bla*_TEM−1B_, *bla*_OXA-10_, and *bla*_CTX-M-65_ were three major acquired extended-spectrum β-lactamase-encoding genes. A total of 32 types of plasmid replicons were detected, with IncQ1 being the most abundant, followed by IncHI2/IncHI2A and IncR. The highest antibiotic resistance rates were identified among *S.* Kentucky isolates in both acquired resistance genes and chromosomal point mutation in the quinolone-resistance-determining regions. Compared with ST19 *S.* Typhimurium, *S.* Typhimurium monophasic variant strains belonged to ST34 and exhibited broad antibiotic resistance. Phylogenetic analysis identified a unique clone of monophasic *S.* Typhimurium obtained from humans and animals, suggesting that they may have the same origin and may have gone through the same genetic evolutionary event. This study provides further insights into *Salmonella* characterization and provides a foundation for further scientific research. The inconsistencies between resistance phenotypes and resistance genes need further investigation.

## Figures and Tables

**Table 1:** Antimicrobial resistance for *Salmonella* isolates collected from this study.

Antibiotics	All	*S*. Typhimurium Monophasic Variant (*n* = 84)	*S*. Typhimurium (*n* = 25)	*S*. Kentucky (*n* = 6)	Food Isolates (*n* = 15)
TET	69.3%	96.4%	60.0%	100.0%	53.3%
AMP	73.5%	94.1%	80.0%	100.0%	40.0%
CHL	57.7%	73.8%	56.0%	83.3%	60.0%
SXT	51.2%	64.3%	48.0%	83.3%	40.0%
AMS	39.1%	56.0%	24.0%	50.0%	13.3%
CFZ	30.2%	38.1%	28.0%	100.0%	6.7%
CTX	21.4%	33.3%	8.0%	100.0%	6.7%
GEN	19.5%	25.0%	4.0%	50.0%	26.7%
CIP	15.4%	13.1%	4.0%	100.0%	6.7%
FEP	8.8%	13.1%	8.0%	33.3%	0.0%
CAZ	7.9%	10.7%	4.0%	33.3%	0.0%
NAL	22.3%	8.3%	28.0%	100.0%	13.3%
AZM	8.8%	4.7%	8.0%	33.3%	13.3%
AMC	1.4%	1.2%	0.0%	0.0%	0.0%
CFX	2.3%	1.2%	0.0%	0.0%	0.0%
CT	4.2%	1.2%	4.0%	0.0%	0.0%
POL	1.9%	1.2%	0.0%	0.0%	0.0%
CZA	0.00%	0.00%	0.0%	0.0%	0.0%
AMI	0.00%	0.00%	0.0%	0.0%	0.0%
IPM	0.00%	0.00%	0.0%	0.0%	0.0%
MEM	0.00%	0.00%	0.0%	0.0%	0.0%
ETP	0.00%	0.00%	0.0%	0.0%	0.0%

**Table 2:** The percentage of MDR among different serotypes.

Serotypes	MDR	Total	Percentage
*S*. Kentucky	6	6	100.0%
*S*. Montevideo	2	2	100.0%
*S*. Muenster	1	1	100.0%
*S*. Kedougou	1	1	100.0%
*S*. Infantis	1	1	100.0%
*S*. Indiana	1	1	100.0%
*S*. IIIb 61:i:e,n,x,z15	1	1	100.0%
*S*. Fanti	1	1	100.0%
*S*. Anatum	1	1	100.0%
*S*. Albany	1	1	100.0%
*S*. Rissen	8	10	80.0%
*S*. Typhimurium monophasic variant	65	84	77.4%
*S*. Derby	3	4	75.0%
*S*. London	10	15	66.7%
*S*. Goldcoast	4	6	66.7%
*S*. Agona	2	3	66.7%
*S*. Typhimurium	14	25	56.0%
*S*. Enteritidis	8	15	53.3%
*S*. Thompson	3	6	50.0%
*S*. Stanley	1	2	50.0%
*S*. Corvallis	0	5	0.0%
*S*. Newport	0	4	0.0%
*S*. Wil	0	3	0.0%
*S*. Give	0	3	0.0%
*S*. Potsdam	0	2	0.0%
*S*. Lichfield	0	2	0.0%
*S*. Braenderup	0	2	0.0%
*S*. Oranienburg	0	1	0.0%
*S*. Virchow	0	1	0.0%
*S*. Saintpaul	0	1	0.0%
*S*. Orion	0	1	0.0%
*S*. Livingstone	0	1	0.0%
*S*. Johannesburg	0	1	0.0%
*S*. Grumpensis	0	1	0.0%
*S*. Bareilly	0	1	0.0%
Total	134	215	62.3%

## Data Availability

The data can be obtained upon request from the corresponding author.

## Supplementary Materials

The following supporting information can be downloaded at: https://www.mdpi.com/article/10.3390/antibiotics13050443/s1, Table S1: Characterizations of *Salmonella* isolates in this study; Table S2: MIC values of *Salmonella* isolates in this study; Table S3: Resistant genes carried in all, *S*. Typhimurium, and *S*. Typhimurium monophasic variant isolates.
