# Supplementary material for: Prevalence and Antimicrobial Resistance Diversity of Salmonella Isolates in Jiaxing City, China
Source: Antibiotics (Basel). 2024 May 14;13(5):443. doi: 10.3390/antibiotics13050443 (PMC11117378; doi:10.3390/antibiotics13050443)
Supplement: Supplementary file 1 [file antibiotics-13-00443-s001.zip › antibiotics-2985221-supplementary/Table S3-new.pdf]

Table S3. Resistant genes carried in all, *S*. Typhimurium, and *S*. Typhimurium monophasic variant isolates

Resistant gene	All isolates (n=215)		*S*. Typhimurium (n=25)		*S*. Typhimurium monophasic variant (n=84)	
*bla*TEM-1B	128	59.5%	19	76.0%	63	75.0%
*flo*R	92	42.8%	13	52.0%	42	50.0%
*sul*2	90	41.9%	14	56.0%	44	52.4%
*qnrS*1	81	37.7%	12	48.0%	44	52.4%
aph(6)-Id	65	30.2%	4	16.0%	37	44.1%
aph(3'')-Ib	65	30.2%	5	20.0%	39	46.4%
tet(A)	58	27.0%	15	60.0%	7	8.3%
**tet*(B)	52	24.2%	0	0	52	61.9%
sul1	41	19.1%	1	4.0%	19	22.6%
aph(3')-Ia	37	17.2%	1	4.0%	21	25.0%
*cml*A1	34	15.8%	9	36.0%	14	16.7%
*aad*A1	32	14.9%	9	36.0%	9	10.7%
*sul*3	31	14.4%	9	36.0%	5	6.0%
*dfrA*12	25	11.6%	9	36.0%	1	1.2%
ARR-3	25	11.6%	1	4.0%	16	19.1%
aph(4)-Ia	25	11.6%	0	0	18	21.43%
aac(6')-Ib-cr	24	11.2%	1	4.0%	14	16.67%
aac(3)-IV	24	11.2%	0	0	17	20.24%
tet(M)	23	10.7%	13	52.0%	2	2.38%
**lnu*(F)	23	10.7%	0	0	18	21.43%
**bla*OXA-10	23	10.7%	0	0	19	22.62%
**dfrA*14	21	9.8%	0	0	14	16.67%
**bla*CTX-M-65	21	9.8%	0	0	16	19.05%
**ARR-2*	21	9.8%	0	0	15	17.86%
*aad*A2	21	9.8%	9	36.0%	1	1.19%
**dfrA*27	20	9.3%	0	0	14	16.67%
**aadA*16	20	9.3%	0	0	15	17.86%
aac(3)-IId	18	8.4%	1	4.0%	3	3.57%
*bla*CTX-M-55	12	5.6%	1	4.0%	6	7.14%
mph(A)	10	4.7%	2	8.0%	0	0
*ble*O	9	4.2%	4	16.0%	4	4.76%
*qnr*B6	8	3.7%	0	0	3	3.57%
*fos*A7	8	3.7%	0	0	0	0
*catB*3	6	2.8%	1	4.0%	2	2.38%
*aadA*2b	6	2.8%	0	0	4	4.76%
*bla*OXA-1	5	2.3%	1	4.0%	2	2.38%
cml	4	1.9%	0	0	0	0
*cat*A2	4	1.9%	0	0	1	1.19%
*bla*CTX-M-14b	4	1.9%	0	0	0	0
*aadA*22	4	1.9%	1	4.0%	2	2.38%
*qnrS*2	3	1.4%	0	0	1	1.19%

*bla*CTX-M-14	3	1.4%	1	4.0%	2	2.38%
*aadA*8b	3	1.4%	0	0	0	0
*aadA*7	3	1.4%	0	0	0	0
*aadA*17	3	1.4%	0	0	1	1.19%
aac(3)-Id	3	1.4%	0	0	0	0
*qnr*B4	2	0.9%	1	4.0%	0	0
qacE	2	0.9%	0	0	1	1.19%
msr(E)	2	0.9%	0	0	0	0
mph(E)	2	0.9%	0	0	0	0
*fosA*3	2	0.9%	0	0	2	2.38%
erm(B)	2	0.9%	1	4.0%	0	0
*dfrA*1	2	0.9%	0	0	0	0
*bla*LAP-2	2	0.9%	0	0	1	1.19%
*bla*CTX-M-24	2	0.9%	1	4.0%	1	1.19%
aph(3')-IIa	2	0.9%	0	0	0	0
tet(G)	1	0.5%	0	0	0	0
tet(D)	1	0.5%	0	0	1	1.19%
rmtB	1	0.5%	1	4.0%	0	0
*qnrB*19	1	0.5%	0	0	0	0
*Oqx*B	1	0.5%	0	0	1	1.19%
*Oqx*A	1	0.5%	0	0	1	1.19%
mcr-1.1	1	0.5%	0	0	1	1.19%
*dfrA*17	1	0.5%	0	0	0	0
*bla*TEM-1A	1	0.5%	0	0	0	0
*bla*OXA-16	1	0.5%	0	0	1	1.19%
*bla*DHA-1	1	0.5%	0	0	0	0
*bla*CARB-2	1	0.5%	0	0	0	0
*aadA*5	1	0.5%	0	0	0	0
*aadA*3	1	0.5%	0	0	0	0
aac(6')-Iaa	1	0.5%	0	0	0	0
